# Hypatia

**Published:** 1888-05

**Authors:** 


### HYPATIA.

Alexandra was, as is well known, at one time the great seat of learning, the intellectual capital of the whole world. It will always be celebrated as the birth-place of some of the noblest and greatest of mankind. In Alexandria was born, in the year 370, Hypatia, daughter of Theon, the famous mathematican of that period. He educated his daughter in mathematics and philosophy, taking great pains with her training. Little is known of her mother; but we infer that she must have been a woman of noble qualities to be the parent of such a daughter as Hypatia who was of that high order of intellect which absorbs knowledge readily, grasping and retaining all that came in her way.

She was sent to Athens in her early days, and studied under the Neo-Platonist, Plutarch, who taught philosophy and expounded the ancient oracles of Chaldea. Under his instruction and guidance she examined theurgy, the supposed art of communicating with the gods. Finishing her course of study, she returned to Alexandria, a lovely, highly-educated and most accomplished woman.

She was one of the most beautiful of her sex, witty eloquent, and possessing the most pleasing, gentle, and attractive manners. With all her wit, beauty and eloquence, she was neither vain, haughty, nor self-conceited. She became a teacher, reopening the school of Plotinus, and soon became surrounded by a class of students, eager to learn of one so gifted and learned in the sciences.

She was called the "Mistress of Philosophy," and became the literary belle of Alexandria. Undoubtedly, she was the best educated woman of her time, and drew around her the *savants* and wise men of the city and surrounding country, among them was the wise and learned Orestes, Governor of Alexandria, who often consulted with her, and deferred to her opinions in State matters. Naturally a woman of her beauty and rare abilities had many admirers, and many lovers sought her hand in marriage. But she turned from them all, and resolved to devote her time and talents to teaching philosophy, and mathematics.

It was unusual for a woman to teach in those days; and, as she did not accept the teachings of Christianity, she provoked the animosity of those Christians who were then in power. They declared she rebelled against the authority of St. Paul, as she did, for Paul had said a woman should not teach, but should sit in silence and subjection. Because Hypatia disregarded this edict, and was a Pagan in religion, she became an object of spite to the bigots of the Church. Not content with ridiculing her and applying to her the most opprobrious and false epithets, they resolved to put her to death, because she taught, among other things, that monasticism was silly and ridiculous.

Hypatia, notwithstanding the threats made against her person, pursued the even tenor of her way, studying and teaching every day. Cyril, who was, at that time, Bishop of Alexandria, instructed his followers to silence the famous teacher, and told them what to do to carry out a most diabolical plan. One day, as she was returning from the academy where she taught, she was seized by a party of monks, dragged from her carriage, and stripped naked in the public streets. They then forcibly carried her fainting form into a church, which was called Cæsareum, and there murdered her, scraplng the flesh from her bones with sharp shells. After this was done they took her bones to a place called Cinaron, and burnt them.

It was as if Philosophy was struck dumb at the martyrdom of this lovely and eloquent expounder of its doctrines. Literature was mangled with her sweet and attractive person, and lay prostrate through that night of woe and despair appropriately called the "Dark Age." The indignation of her followers was so great at this cruel and most dastardly treatment of Hypatia, that Christianity afterwards changed the story of her horrible death, to make it appear that she perished in the cause of religion. And in the Catholic Church to-day, she is numbered among the saints, known to the followers of that religion as Saint Catherine of Alexandria.—*Secular Review.*

